# Peer Review of “Sexual Health Assessment Is Vital to Whole Health Models of Care”

**DOI:** 10.2196/40301

**Published:** 2022-07-28

**Keywords:** sexual health, sexual health assessment, veteran, health equity, health assessment, whole health model, communication, communication barrier, technological barrier, health care, sexuality, sexual orientation, gender identity, sex, gender, model, care, barrier, well-being, comfort, assessment, EHR, electronic health record, quality, equity

*This is a peer-review report submitted for the paper “Sexual Health Assessment Is Vital to Whole Health Models of Care.”*

## Round 1 Review

### General Comments

Thank you for the opportunity to review this manuscript [1] entitled: Sexual Health Assessment as Part of a Whole Health Model to Care: Improving Communication and Technological Barriers. This review paper aims to summarize barriers to assessing sexual health and to suggest some ways to overcome them. The paper summarizes sexual health issues from a new perspective (whole health model), trying to draw some practical new methods. However, several significant questions should be explained before publication.

### Specific Comments

#### 1. Abstract

This seems to be a review article in my understanding.

I suggest reorganizing the abstract.

For review articles, the abstract specifies the topic of the review and the main conclusions drawn.

Additionally, why is there a holistic health model in the title and no mention of it in the abstract?

Also, why the Veterans Health Administration (VHA) is specifically mentioned needs to be answered in the abstract.

Finally, the purpose of the review needs to be reclarified in the abstract. As of the current version, the purpose of this paper seems to be reported too vaguely. Is the authors’ main intent to provide recommendations for development and implementation for the VHA through this review or is the target population just veterans?

#### 2. Introduction

It is recommended to reorganize in a logical order.

Similar to the summary, readers are confused by the sudden mention of veterans.

Since the first paragraph throws out the concept of sexual health. I propose to consolidate the second and third paragraphs while reducing the content. First, mention the fact that sexual health is now an integral part of overall health, then introduce the benefits of achieving sexual health (the original two or three paragraphs), and finally the current obstacles to achieving sexual health (original fourth paragraph).

#### 3. Barriers to Assessing Sexual Health

I personally recommend independent secondary headings.

Of the three obstacles mentioned in the paper, the first is too long to describe. I suggest points 2 and 3 need to be longer to make the structure of the paper smoother.

#### 4. Benefits of Assessing Sexual Health

I personally recommend independent secondary headings here also.

Assessing the benefits of sexual health highlights the importance of sexual health, which is the point of the entire review. I think this paragraph should be moved to the front.

#### 5. Overcoming Barriers to Sexual Health Assessment

I think patient education is a key point in achieving sexual health assessment and needs to be covered in detail.

#### 6. Conclusion

I think the conclusion of the current version needs to be reorganized.

In short, if it is too long it leads to a loss of readability. The conclusion needs to be concise and outline what the paper does exactly, such as what problems were found and what solutions were proposed.

## Abbreviations

VHA: Veterans Health Administration
